# The effect of exercise training intervention for patients with abdominal aortic aneurysm on cardiovascular and cardiorespiratory variables: an updated meta-analysis of randomized controlled trials

**DOI:** 10.1186/s12872-024-03745-x

**Published:** 2024-01-30

**Authors:** Qi Han, Li Qiao, Li Yin, Xuemei Sui, Wenjuan Shao, Qirong Wang

**Affiliations:** 1 Sports Nutrition Center, National Institute of Sports Medicine, Beijing, 100029 China; 2 Beijing Competitor Sports Nutrition Research Institute, Beijing, 100029 China; 3 Department of Vascular Surgery, The Second Affiliated Hospital of Zhejiang University, School of Medicine, Hangzhou, 310020 China; 4 Department of Exercise Science, Arnold School of Public Health, University of South Carolina, Columbia, SC 29208 USA; 5 Beijing Sport University, Beijing, 100084 China; 6 Minzu University of China, Beijing, 100081 China; 7 Department of Surgery, Northwestern University, Chicago, IL 60611 USA

**Keywords:** AAA, CVD, Exercise, Rupture risks

## Abstract

**Objective:**

The purpose of this meta-analysis was to evaluate the effect of exercise training intervention in patients with abdominal aortic aneurysm (AAA).

**Methods:**

Eight randomized controlled trials (RCTs) that recruited 588 AAA patients were extracted using 4 databases (PubMed, Embase, Wanfang Data, and Cochrane Library). Physiological and biochemistry parameters that included in this study are high-sensitivity C-reactive protein (hs-CRP), respiratory peak oxygen uptake rate (VO_2_peak), triglyceride (TG), total cholesterol (TC), anaerobic threshold (AT), the diameter of AAA, high density lipoprotein cholesterol (HDL), low density lipoprotein cholesterol (LDL), and matrix metalloproteinase-9 (MMP-9). Standard mean difference (SMD) was used to assess the between group effect.

**Results:**

This meta-analysis was synthesized with findings from RCTs and found that hs-CRP (SMD, − 0.56 mg/dL; 95% CI: − 0.90 to 0.22; *P* = 0.001), VO_2_peak (SMD, 0.4 mL/kg/min; 95% CI, 0.21 to 0.60; *P* < 0.001), TG (SMD, − 0.39 mg/dL; 95% CI: − 0.02 to 0.77; *P* = 0.04), and AT (SMD, 0.75 mL/kg/min; 95% CI, 0.54 to 0.96; *P* < 0.001) were significantly improved in the exercise groups, while the size of AAA (SMD, − 0.15; 95% CI: − 0.36 to 0.06; *P* = 0.15), TC (SMD, 0.16 mg/dL; 95% CI: − 0.10 to 0.42; *P* = 0.23), HDL/LDL ratio (SMD, − 0.06; 95% CI: − 0.32 to 0.20; *P* = 0.64), HDL (SMD, − 0.09; 95% CI: − 0.39 to 0.20; *P* = 0.54), LDL (SMD, 0.08; 95% CI: − 0.21 to 0.38; *P* = 0.59), and MMP-9 (SMD, − 0.23 mg/dL; 95% CI: − 0.53 to 0.06; *P* = 0.12) did not differ in the exercise groups compared with the controls.

**Conclusion:**

Exercise intervention improved some of the CVD risk factors but not all, hs-CRP, VO_2_peak and AT were significantly improved after exercise intervention, while, changes of MMP-9, the size of AAA, and the overall lipids profile were not. Exercise intervention provides an additional solution for improving cardiorespiratory capacity and health status among AAA patients, and might lead to a delay of AAA progression.

## Strength and limitations of this study

The strength of this study is that the level of evidence synthesized by meta-analysis is on the top of evidence-based pyramid for clinical research. Compared to single RCTs, case-control studies, cohort studies, opinions, our meta-analysis provide tier 1 evidence because it included most up-to-date peer-reviewed RCTs and excluded cohort studies, control trials without randomization, case-control studies, as well as descriptive cross-sectional observations.

The limitations are that only small sample sizes of the trials can be included, and the fact that AAA patients in the studies were from diverse characteristics. With limited number of RCTs, it was therefore not feasible to adjust for all different variables in the subgroups, such as mode of exercise training, age, gender, etc.

## Introduction

Abdominal aortic aneurysm (AAA) usually occurs in the infrarenal part of the aorta, and is usually accompanied by a variety of risks for rupture and sudden death [1–3]. AAA generally happens and forms below the aortic hiatus of the diaphragm to the point of bifurcation [1]. Its formation closely associates with chronic aortic inflammation, increased local expression of proteinase, and degradation of connective tissue protein [4]. AAA is clinically diagnosed as the maximal aortic diameter above 3 cm that is measured in any plane, which is perpendicular to the artery vessel axis [5–7]. AAA expands gradually over a period of time, and can sometimes rupture before death. More than 15,000 deaths per year occur in the US related to AAA rupture and its complications after surgery [8, 9]. Death from a ruptured AAA can occur before it is diagnosed or reported, and therefore the actual mortality rate of AAA can be underestimated [9]. In practice, regular health screening and examination of small AAA could be of great importance for the management and overall survival rate of AAA [10–12]. Wilmink AB et al. [13] reported that screening for asymptomatic AAA can reduce the incidence of its rupture, and elective repair of the asymptomatic lesion save lives.

AAA associates with multiple risk factors [14]. Males over 60 years old are considered to have higher risks than females, and the prevalence of unsuspected, asymptomatic AAA in men older than 60 is 4 to 8%, while in women it is 0.5 to 1.5% [8]. Asian males are less likely to suffer AAA than White males, and therefore ethnicity is also considered to be a risk factor [15]. Smoking, family history, cardiovascular disease (CVD), coronary artery disease (CAD), hyperlipidemia, hypertension, and diabetes have also been reported to be risk factors for AAA formation and progression [16–18]. Besides the evidence-proved and well-recognized clinical risk factors for AAA, it is still under debate whether a single elevation of CVD or CAD risk factor can be positively correlated with AAA progression. Nakayama reported in their study that although CAD was widely acknowledged to be co-existent with AAA, they found an inverse association between CAD existence and the AAA expansion rate [19]. Takagi found that CAD is associated with AAA growth under fixed-effect quantitative synthesis model (SMD, − 0.06; 95%CI, − 0.12 to − 0.00; *P* = 0.04), however, their result was not significant under random-effects statistic model (SMD, − 0.06; 95%CI, − 0.13 to 0.01; *P* = 0.12) [20]. Avdic and Lederle both found that patients diagnosed and under treatment of type 2 diabetes mellitus (T2DM) demonstrated reduced risk of AAA compared to health control [21, 22]. In accordance, Climent also reported that drugs used to treat T2DM showed protective effect on the expansion of AAA compared to non-T2DM group [23]. There can be several reasons for the inverse association between AAA and CAD existence, which possibly due to the use of certain medication (e.g. ACEi [24], β-blockers [25], statins [26]) to treat CAD that slowed or attenuated the expansion rate of AAA compared to their control group who did not use medication. While, in this meta-analysis, we want to find the effect of exercise intervention on AAA and certain CVD risk factors regardless of their use of CAD or CVD medication, therefore, the confounding effect of use CAD or CVD medication intervene wasn’t taken into account in our current meta-analysis, the involved participants are expected to keep their regular medication along with their exercise training.

No specific medication was found for significantly reducing the size of AAA, and surgery remains the most important intervention for fast-expanding AAA in clinic [7, 27]. There is usually a catastrophic loss of blood from the site of ruptured AAA before patients reach the emergency room (ER), and some patients do not survive before they reach the ER, which makes the predicted overall mortality rate for AAA lesion-related complications as high as 90% [28–30]. For AAA patients with a maximal aortic diameter from 3.0 to 4.0 cm, and the rate of AAA growth less than 10% per year, annual imaging with abdominal ultrasound is recommended [31–33]. Patients with small AAA, which is between 3.0 cm and 5.5 cm in maximum aorta diameter, are generally treated with medication to reduce the rate of AAA expansion [34]. Open aortic aneurysm surgery repair and endovascular abdominal aortic aneurysm repair (EVAR) are surgical procedures suggested for AAA patients with a maximal aortic diameter greater than 5.5 cm for males and 5.0 cm for females, or AAA patients with any maximum aortic vessel diameter but having predictable fatal lesion and rupture risk [31, 35]. Furthermore, the predicted post-surgery survival length of life is another factor for AAA patients and doctors to consider, based on the age of the patient [36].

It is critical for health-care providers to seek alternative strategies to improve the quality of life among AAA patients. There are growing research focused on non-surgical management of AAA at its early formation, the basic and most comprehensive idea of the treatment is to slow down the dilation of AAA, including the change of dietary patterns, the changes of lifestyle, and applying physical exercise [37]. Pre-operative exercise therapy (PET) has beneficial effects on various physical fitness variables of patients with AAA, e.g. applying moderate intensity exercise training increased exercise capacity (time to exhaust), metabolic equivalents (MET), anaerobic threshold (AT), peak oxygen consumption rate (VO_2_peak), and ventilatory threshold (VT) among AAA patients [38]. A recent meta-analysis evaluated the safety of exercise training and its effect on cardiorespiratory variables in AAA patients, and their meta-analysis showed that although exercise training did not attenuate the progression of the diameter of AAA, it decreased high-sensitivity C-reactive protein (hs-CRP), increased AT as well as VO_2_peak, [39] and they indicated that moderate intensity exercise is generally safe during the exercise tests and training with no ruptures or lesions reported. It is widely acknowledged that appropriate exercise training and stay physically active can help people and CVD patients to maintain adequate skeletal muscle mass, cardiorespiratory function, build immune function, neuromuscular motor control, and mental health [40, 41]. And all the above health benefits from appropriate exercise applies to AAA patients [42]. Adopting exercise strategies with appropriate intensity, to be specific, moderate training intensity is commonly used for patients diagnosed with AAA, and can be helpful to enhance cardiovascular capacity and promote the quality of life for AAA patients.

## Objectives

In this meta-analysis, we aimed to further examine the health benefit of exercise training intervention among preoperative AAA patients on cardiovascular and cardiorespiratory variables.

## Methods

### Design

This meta-analysis was prepared and conducted in accordance with the PRISMA (Preferred Reporting Items for Systematic Reviews and Meta-Analysis) statement [43] and in accordance with PICOs requirements to ensure rigorous methodology and reporting. Prospero ID (CRD42019131199) was registered before data extraction.

### Eligibility criteria

The PICOs approach was used as follows: Population (P) was defined as male and female AAA patients with no severe complications that restricted exercise. Intervention (I) was exercise intervention. Comparison (C) was between intervention and placebo. Primary outcomes (O) were the size/diameter of AAA and some risk factors of CVD indicating rupture risks of AAA. The secondary outcome was cardiorespiratory capacity indicators.

Only RCTs were included, in which the exercise group should have received exercise training and the control group should have received regular and usual care without structured exercise training. The eligibility criteria were set to target all trials conducted in non-operative and pre-operative AAA patients (maximum aortic diameter greater than 3.0 cm) and that included an exercise training intervention. Non-randomized trials, studies without full text, non-AAA trials, and studies that did not address the size of AAA were excluded. Research was also excluded if it did not report sufficient information on study quality, incomplete outcomes, deterioration in cardiac function, or ability of participants to perform exercise. Two reviewers reviewed all eligible trials and determined whether they fulfilled the selection criteria. Disagreements were resolved by discussion and consultation with help from a senior scientist.

### Search methods for identification of articles

A literature search was conducted of PubMed, Embase, Wanfang Data, and Cochrane Library databases from inception to Dec 13th, 2023. The following terms and medical subject headings (MeSH) were searched: (abdominal) AND (aorta OR aortic) AND (aneurysm OR aneurysms) AND (exercise OR interval training OR resistance training OR weight training OR physical fitness OR rehabilitation OR cardiorespiratory fitness OR oxygen consumption OR ventilatory threshold OR anaerobic threshold) AND (randomized OR randomly OR randomization OR randomized controlled trial). Duplicates were then removed at the stage of title and abstract assessment with assistance from Mendeley tools.

This article was prepared in accordance with the Declaration of Helsinki. No ethical approval was required since this study did not involve an intervention on human subjects.

### Eligibility assessment, study selection and quality assessment

The Cochrane risk of bias (RoB) assessment tool was used to screen, select, and assess the quality of trials. Studies were screened by the PRISMA checklist. Titles and abstracts were reviewed for eligibility by two authors independently. Then, two reviewers independently assessed the full text of included articles and their methodological quality, outcomes, and duplication. Disagreements were resolved through consensus.

### Data extraction

Data were extracted independently by two authors. Disagreements were resolved through consensus. Age, gender, nationalities, mode of sports activities, exercise intensity and volume, diameter of AAA, total cholesterol (TC), triglyceride (TG), high-density lipoprotein cholesterol (HDL), low-density lipoprotein cholesterol (LDL), hs-CRP, respiratory peak oxygen uptake rate (VO_2_peak), anaerobic threshold (AT), and matrix metalloproteinase-9 (MMP-9), and outcome measures (mean, SD, unit) were extracted. Standard deviation (SD) was extracted from range, standard errors, and confidence intervals (CIs) if SD was not reported.

When data of interest was missing or could not be extracted, we contacted the original study authors directly by email and phone calls.

### Data stratification and subgroups

Quantitative synthesis considering different outcomes was generated for an overall effect in the analysis of combined effect regarding lipid profile.

### Data synthesis

Heterogeneity was tested using the Cochran’s Q test with *p*-value set at 0.05 for significance and quantified using the I^2^ statistic (I^2^ < 40% as low, 40–60% as moderate, and > 60% as substantial heterogeneity). Review Manager 5.3 was used for data analysis. For within-group mean changes between baseline and follow-up check, we performed mean differences (MD) check and synthesized the SD of the MD. In the forest plot of this meta-analysis, the mean of the exercise group and control stands for the MD between each follow-up check and the baseline assessment. For the between-group effect, we performed standard mean differences (SMD) check using the random-effects model with continuous outcome measurement and in an inverse variance approach.

### Patient and public involvement statement

This research is a meta-analysis study following recommended PRISMA and PICOS study procedure for patient involvement, and it was not possible to involve patients or the public in the design, or conduct, or reporting, or dissemination plans of our research.

## Results

### Eligibility assessment and article selection

**Fig. 1:**
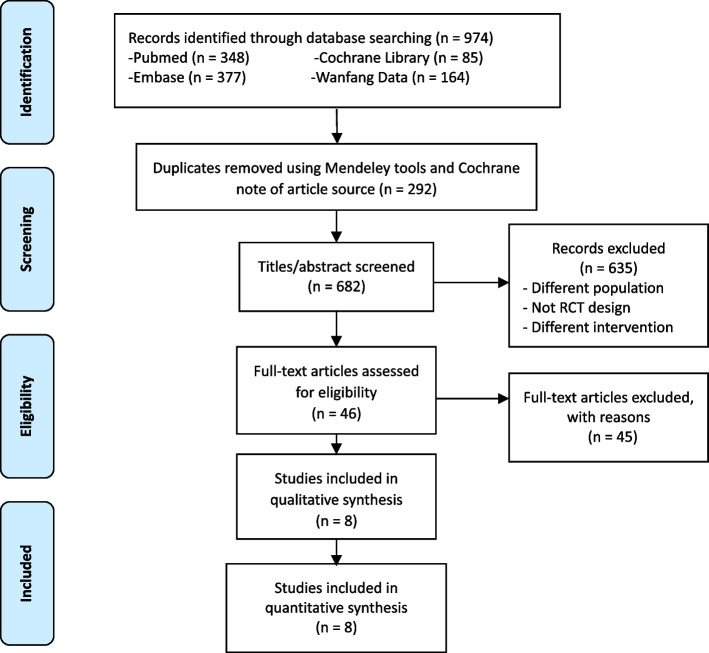
PRISMA flow diagram of the search and selection process

Figure 1 presents the search and selection process. After reviewing 682 titles and abstracts, 46 articles were selected for full-text article review (Fig. 1). Of the 46 articles, eight RCTs [44–51] were included, with a total of 588 AAA patients randomly divided into exercise training group (*n* = 291) or control group (*n* = 297).

No additional studies were found when we manually performed searches with the major databases by the time of submission. All studies included used randomization strategies to ensure that the comparison between exercise training group and control group would provide rigorous evidence among AAA patients. A study from Nakayama was excluded because it did not apply a proper randomization strategy in the prospective pilot study design [52].

### Risk of bias assessment

**Fig. 2:**
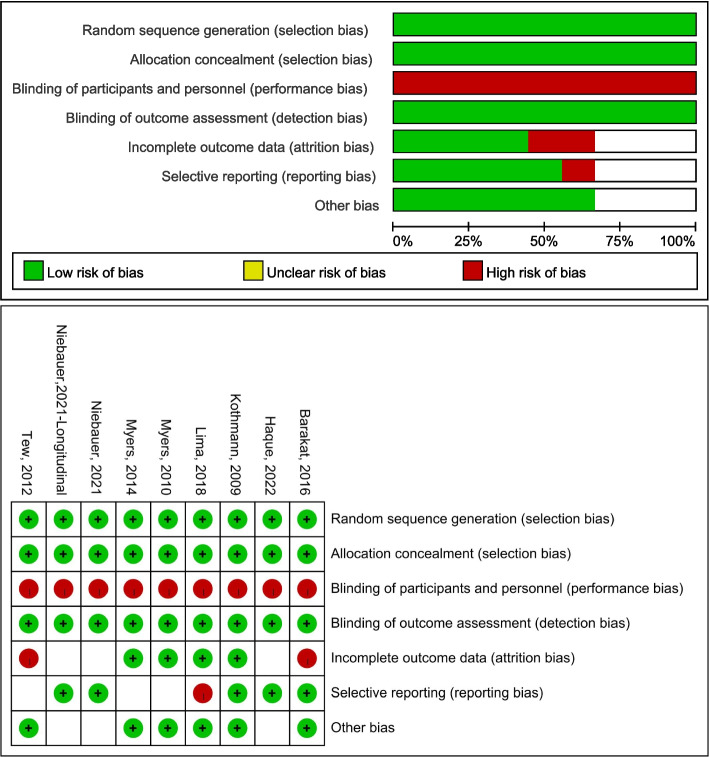
Cochrane risk of bias assessment

Figure 2 is the risk of bias summarized using the Cochrane risk of bias (RoB) tool (Fig. 2). Because of the nature of exercise intervention studies, participants cannot be blinded at the stage of supervised exercise training intervention. Therefore, we rated the blinding of participants and personnel (performance bias) of all included RCTs as a high risk for consistency. Different comparisons for one CVD biomarker from the same study were listed separately, e.g. the AAA diameter changes in two directions reported by Niebauer [50].

### Trials and baseline characteristics

The baseline characteristics of the included studies are presented in Table 1. The sample size ranged from 25 to 140, and their mean age, body mass index (BMI), and AAA diameter were from 70 to 73 years, 27.5 to 28.1 kg/m^2^, and 30 to 62 mm, respectively. The proportion of males ranged from 80 to 100%. At baseline, mean VO_2_peak and AT ranged from 16.1 to 20.2 mL/kg/min and 10.5 to 14.6 mL/kg/min, respectively (Table 1). Barakat et al. [47] included patients with large AAA (above 55 mm) who were scheduled for surgery. To be noticed, although the inclusion criteria did not set the training workload, we observed that all the included RCT studies applied moderate training intensity, which provide very important indication for health-care providers.

**Table 1:** Characteristics of studies included in this research

	Total No.	Age	Male	BMI	AAA diameter	VO_2_peak	AT	Time	Frequency	Duration		
Author	(E/C)*	years	%	kg/m^2^	mm	mL/kg/min	mL/kg/min	min/session	times/wk	weeks	Intensity	Mode
Myers, 2010 [44]	57 (26/31)	71 ± 8	93	27.5 ± 3.9	30–50	20.2 ± 7.2	N/A	55	3	48	Mod	ET + RT
Tew, 2012 [45]	25 (11/14)	73 ± 7	84	28.1 ± 3.2	40 ± 7	18.5 ± 5.1	12.5 ± 3	45	3	12	Mod	ET
Myers, 2014 [46]	140 (72/68)	72 ± 7	92	28.1 ± 3.7	34 ± 5	19.7 ± 6.1	14.6 ± 4.7	55	3	48	Mod	ET + RT
Barakat, 2016 [47]	124 (62/62)	73 ± 7	90	27 ± 3.9	62 ± 8	17.5 ± 4.5	12.5 ± 3.9	60	3	6	Mod	ET + RT
Lima, 2018 [48]	65 (33/32)	72 ± 7	100	28 ± 3.3	37 ± 5	19.2 ± 5.2	14.4 ± 4.2	55	3	12	Mod	ET + RT
Kothmann, 2009 [49]	25 (17/8)	70 (61–79)	80	N/A	40 (30–51)	N/A	10.5 ± 2	40	2	7	Mod	ET
Niebauer, 2021 [50]	96 (42/54)	73 (53–86)	91	26.9–29.2	35–39	N/A	N/A	60	3	48	Mod	ET + RT
Haque, 2022 [51]	56 (28/28)	72.8 ± 5.7	86	27.7 ± 3.6	38 ± 5	16.6 ± 5.1	10.2 ± 2.3	45–60	3	36	Mod	ET + RT

* *E* exercise, *C* control. *BMI* body mass index, *VO*_*2*_*peak* maximum oxygen consumption; *AT* anaerobic threshold, *Mod* moderate, *ET* endurance training, *RT* resistance training

### AAA diameter changes before and after exercise training

**Fig. 3:**
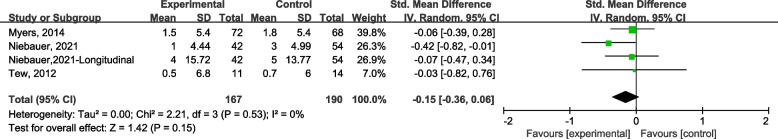
Forest plot for the size of AAA changes

Three studies included assessment of AAA diameter both before and after the intervention. Though promising, from the quantitative synthesis in our meta-analysis, we observed that there was no significant overall effect of exercise on the size of AAA (SMD: -0.15; 95% CI: − 0.36 to 0.06; *P* = 0.15) (Fig. 3). Tew et al. [45] reported that the SMD of AAA diameter was − 0.03 mm (95% CI: − 0.82 to 0.76) over 12 weeks, a negative SMD value of which stands for slower growth of AAA observed in exercise group than control, indicating that the exercise training intervention group did not increase AAA diameter as much as the control over the intervention period. In addition, Myers et al. [46] reported that the annual SMD of AAA diameter was − 0.06 mm (95% CI: − 0.39 to 0.28) in the exercise training group compared to the control group, which indicates that the exercise training intervention group did not increase AAA diameter as much as the control. There was no significant change in maximal longitudinal AAA diameter, while Niebauer et al. [50] reported that transverse diameter significantly increased in both exercise (*P* = 0.012) and control group (*P* = 0.0001).

### Lipid profile of blood biochemistry test

**Fig. 4:**
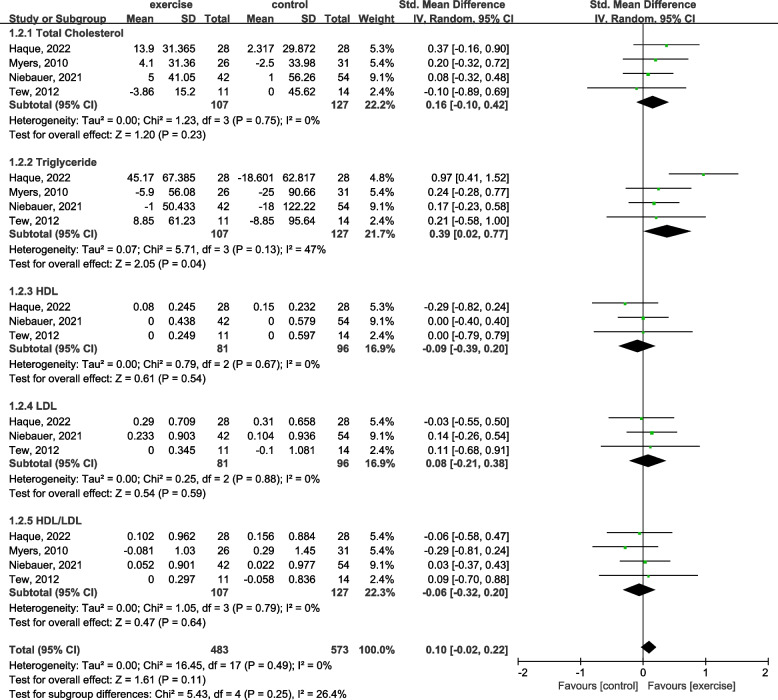
Forest plot for lipid profile of blood biochemistry test outcome changes

TC, TG, HDL, LDL, and HDL/LDL ratio were monitored in four studies [44, 45, 50, 51]. From the forest plot in Fig. 4, the effect of exercise training on TC was SMD = 0.16 mg/dL (95% CI: − 0.10 to 0.42; *P* = 0.23), on TG was SMD = 0.39 mg/dL (95% CI: 0.02 to 0.77; *P* = 0.04) favors exercise, and on LDL was SMD = 0.08 mg/dL (95% CI: − 0.21 to 0.38; *P* = 0.59) compared with the control group, and they showed a positive effect of exercise training. However, the effect of exercise training on HDL was SMD = − 0.09 mg/dL (95% CI: − 0.39 to 0.20; *P* = 0.54), and on HDL/LDL ratio was SMD = − 0.06 (95% CI: − 0.32 to 0.20; *P* = 0.64) compared with the control group, and these results were in favor of the control group. Overall, the adaptation of exercise training to improve lipid profile among AAA patients might be promising, but the effect was not significant (SMD = 0.10 mg/dL; 95% CI: − 0.02 to 0.22; *P* = 0.11) compared with the control (Fig. 4).

### Hs-CRP before and after exercise training

**Fig. 5:**
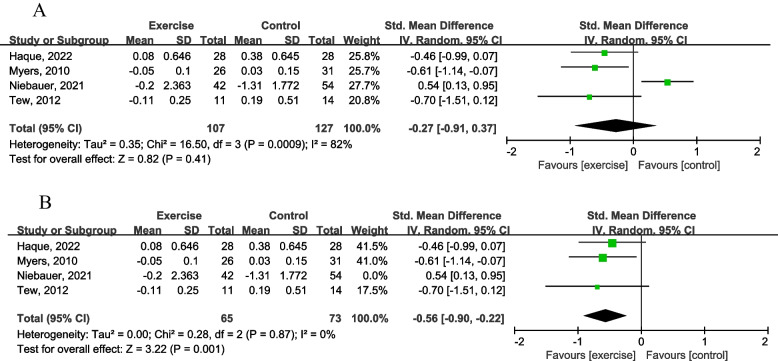
Forest plot for hs-CRP changes. Figure 5A is before reducing the heterogeneity, and Fig. 5B is after reducing the heterogeneity by removing one study from Niebauer

hs-CRP was monitored in four studies [44, 45, 50, 51]. From the forest plot in Fig. 5, the effect of exercise training on hs-CRP compared with the control group was SMD = − 0.27 mg/dL (95% CI: − 0.91 to 0.37; *P* = 0.41) when all four hs-CRP reporting RCT studies were included, and the heterogeneity was large (I^2^ = 0.82) (Fig. 5A). After the exclusion of a study from Niebauer [50], the overall heterogeneity decreased to 0% (Fig. 5B).

### MMP-9 before and after exercise training

**Fig. 6:**
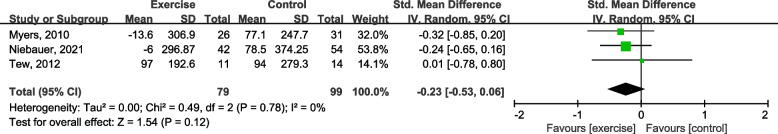
Forest plot for MMP-9 changes

MMP-9 was monitored in three studies [44, 45, 50]. The effect of exercise training on MMP-9 was SMD = − 0.23 mg/dL (95% CI: − 0.53 to 0.06; *P* = 0.12) compared with the control, as shown in Fig. 6. The analysis showed that exercise training might have attenuated the up-regulation of MMP-9. However, the outcome was not significant (Fig. 6).

### Cardiopulmonary test before and after exercise training

**Fig. 7:**
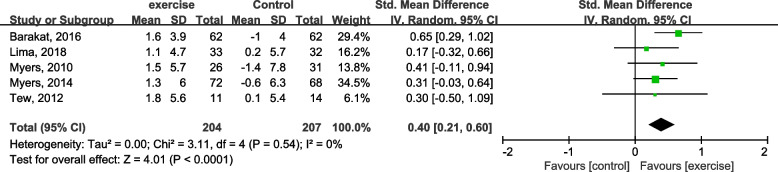
Forest plot for VO_2_peak changes

VO_2_peak was monitored in five studies [44–48]. The effect of exercise training on VO_2_peak among AAA patients in the exercise training group compared with control was SMD = 0.4 (95% CI: 0.21 to 0.60; *P* < 0.0001), as shown in Fig. 7. This finding suggests that exercise training elevated VO_2_peak compared with the control group (Fig. 7).

**Fig. 8:**
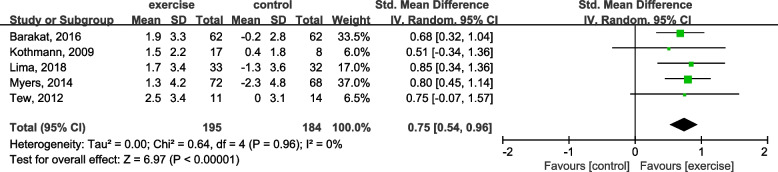
Forest plot for AT changes

AT was monitored in five studies [45–49]. The effect of exercise training on AT among AAA patients in the exercise training group compared with control was 0.75 (95% CI: 0.54 to 0.96; *P* = 0), as shown in Fig. 8. This finding suggests that exercise training elevated AT compared with the control group (Fig. 8).

## Discussion

### Summary of main findings

Exercise training is very important in the recovery process of many surgeries. It not only helps patients regain their physical strength, range of motion, cardiorespiratory and cardiovascular fitness, but also improves their overall health and well-being. Exercise training helps patients manage pain and discomfort during the recovery period, relief the feeling of pain, improve quality of life, and help patients better cope with the difficult times of peri-operation period. And its underlying mechanisms include that exercise can promote the release of endorphins, which was reported to be able to elevate pain perception threshold [53], downregulate TGF-β pathway related genes expression, normalize inflammation-related genes expression including PPARs, BCL-2α1, and VCAM-1 [54]. After surgery, many patients experience respiratory complications such as pneumonia or short of breath. Cardiorespiratory exercise can help improve lung capacity and reduce the risk of these complications. Patients may also experience changes in their appetite and metabolism after surgery with the use of corticosteroids, which can lead to weight gain. Exercise helps regulate appetite and energy expenditure, facilitating the process of returning to a healthy weight if necessary [55].

In this current meta-analysis, we found that exercise intervention improved some of the CVD risk factors but not every, hs-CRP, VO_2_peak, and AT were significantly improved after exercise intervention, while, changes of MMP-9, the size of AAA, and the overall lipids profile were not. Exercise intervention provides an additional solution for improving cardiorespiratory capacity and health status among AAA patients [46], and might delay its progression [50, 56]. All the articles that met our inclusion criteria applied moderate intensity exercise in the exercise intervention, and moderate intensity training can be accepted by the majority of AAA patients without adverse events during their training and cardiorespiratory exercise testing. Meanwhile, because most AAA patients are suffering from the atherosclerosis, hardening of the arteries, inflammation, and plaque, an ideal exercise training program shall be supervised and guided by a medical doctor, athletic trainer, personal trainer, or AHA certified CPR & first aid personnel. The key point to always keep in mind is that AAA patients shall avoid sudden high volume and high intensity training, and the sudden dilation of the arteries might increase the risk of aneurysm lesion and even rupture. Therefore, keep moderate intensity of training and seek adequate supervision always comes on the top with the highest priority for AAA patients.

#### Size of AAA

The size of AAA is critical for diagnosing and managing aneurysms [57]. The American Heart Association (AHA) and American College of Cardiology (ACC) [7] have stated that supervised and structured exercise has a strong beneficial effect on the management of CAD. Although no significant overall effect of exercise on the size of AAA (*P* = 0.15) compared with the control group was observed in this study, it cannot be simply interpreted that the exercise intervention did not attenuate the progression of AAA, as only three RCTs were eligible to be included in this meta-analysis. Recent studies also indicated that exercise intervention might [58] or may not [50] be associated with reduced rate of AAA expansion. In the future, peer-reviewed RCTs with more AAA patients are still needed to examine the impact of structured exercise training intervention on the management of AAA with various subgroups.

During exercise, cardiovascular system has increased demand of consuming oxygen and nutrients, including increased cardiac output, stroke volume, blood pressure, and heart rate. This can place considerable stress on the aorta, especially in AAA patients. Therefore, it is also crucial to monitor and manage the haemodynamic changes during exercise to ensure safety, since the aortic aneurysm may not be able to withstand the increased blood pressure and shear stress induced by vigorous physical activity [59]. And patients with AAA should be carefully assessed and supervised to undertake their personalized exercise intensity to minimize the risk of adverse cardiovascular events. Especially for those peri-operative AAA patients at their first inpatient visiting, it is essential to have a comprehensive discussion with their doctor about the safety of exercise prescription, which shall include assessing medical history, current medication, physical fitness, the size of AAA, haemodynamic monitoring with PC-MRI [60, 61], and other healthy issues. Healthcare professionals should also be trained to supervise haemodynamic changes during exercise, ensuring that patients participate in exercise that are safe and optimized to their health promotion objectives.

#### Lipid profile of blood biochemistry test

Multiple cardiovascular disease risk factors can be present in patients diagnosed with AAA, and dyslipidemia plays an important role in the formation and progression of AAA [62]. From this meta-analysis, exercise training significantly improved TG compared to the control (*P* < 0.05). While, the standard mean difference on TC, HDL, and LDL were not significant (*P* > 0.05).

Two major forms of lipoproteins carry cholesterol within the human circulation system. HDL is acknowledged to be good cholesterol, and it has been reported that the progression of AAA can be limited by elevating HDL [63]. LDL is believed to be bad cholesterol, associated with the formation and progression of AAA [64]. In general, cholesterol can be delivered to artery endothelium by LDL, creating risk factors for CVD, while HDL transports cholesterol back to the liver. Low availability of HDL is a risk factor for the progression of cardiovascular disease [65].

Up-regulated lipids or triglycerides level means fat and lipids are accumulated in the blood. An up-regulated TG level can usually be seen as a sign of type 2 diabetes as well as metabolic syndrome, and indicate high risk of CVD, stroke, and heart attack. The elevated exercise energy expenditure and appropriately designed dietary intake contribute to the decrease in TG, LDL, TC levels through the process of mobilization of fat tissue, fatty acid β-oxidation in the mitochondrial matrix, and enhanced Kreb’s cycle (TCA cycle) leads to a reduction in lipid deposition not only in the circulation system, but also in the arterial endothelium tissue, thereby could reduce the risk of arteriosclerosis, atherosclerosis, and aneurysm formation. In specific to the significance of this AAA study, down-regulated TG level may be helpful to the less hardening of the arteries or thickening of the artery walls (less risk for the formation of arteriosclerosis, atherosclerosis, aneurysms, etc.). Therefore, down-regulated TG level indicate less risk of the formation of aneurysm in the aortic blood vessel.

#### Hs-CRP

There are many health benefits by applying exercise training on the status of CVD patients, including help to build better immune function, cardiopulmonary function, vascular smooth muscle cell condition, reactive oxygen species (ROS) status, and even mental health. Improved inflammation status but not worsening inflammation situation can help people attenuate the process of atherosclerosis formation and probably delay the progression of AAA. The adaptation of long-term moderate exercise intervenes attenuated one of the physiological inflammatory biomarkers, hs-CRP, which can indicate less inflammatory stress presented in the circulatory system after exercise training adaptation [66]. Elevated level of CRP, which produced and secreted by the liver, was observed along with AAA formation [67]. The increase of C-reactive protein correlate with the risk of getting coronary artery disease (CAD) or cardiovascular disease (CVD). Furthermore, altered wall shear stress (WSS), high oscillatory shear index (OSI) and high relative residence time (RRT) are correlated with increase CVD inflammatory risk factors of the blood vessel [68]. Increased status of CRP expression in low WSS situation indicates enhanced pathogenesis activity of inflammation in certain condition [69–71]. There is a study from Kojima reported that WSS has been considered a major determinant of atherosclerosis [72]. Another study conducted by Manli Zhou reported that low WSS is the factor of early plaque formation, occurrence and development [73], and a high WSS can promote the transformation of plaque to high-risk phenotype including various CVD [74].

We also analyzed changes of hs-CRP in AAA patients. From the forest plot in Fig. 5B, exercise training significantly improved hs-CRP status compared with the control group. Therefore, this meta-analysis found that exercise training attenuated the up-regulation of hs-CRP (*P* < 0.05) [44, 45, 51].

#### MMP-9

Matrix metalloproteinase-9 (MMP-9) is involved in AAA formations and studies have suggested that down-regulated MMP-9 reduces AAA growth [75, 76]. Treatment and pharmacological medication targeting MMPs, for instance, the use of doxycycline, and 3-hydroxy-3-methyglutaryl coenzyme A reductase inhibitor (cerivastatin) suppress the production of MMPs, were shown to suppress the development of AAA [77–82]. And it was widely acknowledged that long-term exercise training adaptation of appropriate intensity exercise increase eNOS expression, vascular endothelium function, and down-regulate inflammation through decreasing TNF-α, IL-6, MMPs, NFκB contents [83, 84]. However, in this present quantitative synthesis of our meta-analysis, exercise training intervention on the regulation of MMP-9 among AAA patients was not significantly different compared to the control group.

#### VO_2_peak and AT as secondary outcome

From Kato’s recent study, we found that applying moderate intensity exercise on AAA patients is generally safe with no severe adverse event was reported [39]. In this current study, no adverse event had been reported from the studies included in our selected articles for the quantitative synthesis. It was well-acknowledged that long term regular exercise training can improve cardiovascular adaptation, and our quantitative synthesized findings in this meta-analysis also demonstrated that it can be helpful in AAA patients to enhance cardiorespiratory function through elevating VO_2_peak and anaerobic threshold.

Five trials assessed VO_2_peak, with a total of 204 patients in the intervention group and 207 patients in the control group [44–48]. And VO_2_peak of the exercise training group was significantly elevated compared to the control group, with SMD = 0.4 mL/kg/min (95% CI, 0.21–0.60; *P* < 0.001), at the same time, AT investigated from the exercise training groups was significantly enhanced compared to the control, with SMD = 0.75 mL/kg/min (95% CI, 0.54–0.96; *P* < 0.001) [45–49]. These two indexes can indicate enhanced cardiorespiratory capacity and quality of life.

### Overview of overall quality

PRISMA criteria and PICOs procedures of meta-analysis were applied to ensure quality and rigorous methodology. The selection and review process were independently conducted by two authors.

### Strengths and limitations

The strength of this study is that the level of evidence synthesized by meta-analysis is on the top of evidence-based pyramid for clinical research. Compared to single RCTs, case-control studies, cohort studies, opinions, our meta-analysis provide tier 1 evidence because it included most up-to-date peer-reviewed RCTs and excluded cohort studies, control trials without randomization, case-control studies, as well as descriptive cross-sectional observations.

Our study has limitations that cannot be disregarded, such as the small sample sizes of the trials included, and the fact that AAA patients in the studies were from diverse backgrounds with different professions, nationalities, and locations. The proportion of males included in this meta-analysis ranged from 80 to 100%. Although the risk of AAA formation in men is higher than in women, the current meta-analysis results could not be extrapolated to women without a thoroughly investigation in female population.

With the limited number of RCTs, it was therefore not feasible to adjust for all different variables in the subgroups, such as mode of exercise training, age, gender, and use of medication [2] for long-term AAA management (e.g., ACEi, beta-blockers, statins). Although exercise related to increased blood flow velocity, blood vessel dilation, and elevation of shear stress on the aortic artery that might increase the rupture risks of AAA, none of the selected RCTs reported AAA rupture case during moderate intensity exercise intervention or exercise testing, therefore, a zero AAA rupture risk was reported relating to moderate training in this current meta-analysis.

The major outcome of observing no overall effect of exercise training on the MD of AAA diameter changes, combined lipid profile changes, and MMP-9 changes could be due to the small sample size within each outcome measurement and not being able to stratify the AAA patients for better control when pooling and summarizing each outcome.

Though structured exercise was reported to improve overall fitness among CAD patients [7], peer-reviewed RCTs with large samples of exercise training intervention for patients with small or pre-surgical AAA are still needed. For those who undertake endovascular EVAR repair (e.g. surgical bypass, applying AAA stent graft), a preoperative moderate exercise intervention was reported to be positively associated with better post-surgical outcomes [47, 85], including 1) Pre-operative exercise training can enhance cardiorespiratory function [86]; 2) Pre-operative exercise helps to maintain skeletal muscle function, and probably reducing the time of returning to work after surgery [47] regardless of their ICU or hospital stay [87]; 3) Pre-operative exercise might help to maintain appropriate energy expenditure and reduce the risk of renal and cardiac complications after surgery [87]; 4) Exercise has been demonstrated to enhance vascular function through regulating PPARs, ERs, RXR signaling pathways [54, 84], which are targets for pharmacological treatment of patients with AAA; 5) Post-operative exercise has been demonstrated to be associated with reduced CVD risk factors, e.g. improved lipids profile [50], cardio-pulmonary capacity [88], immune system, and blood pressure [89]; 6) Pre-operative supervised exercise could potentially improve the overall mortality rate [90, 91]. On the top of supervised exercise program protocol design, it is essential to tailor exercise according to different cases regarding physical fitness status and medical conditions. Therefore, it is crucial for healthcare providers to emphasize the importance of exercise intensity and provide guidance and supervision to patients throughout the rehabilitation process, and could be our future research directions.

## Conclusions

Nine risk factors of AAA development and progression were included in this meta-analysis, and hs-CRP, TG, VO_2_peak, and AT were significantly improved in the exercise training group, while the size of AAA, TC, LDL, HDL, HDL/LDL ratio, and MMP-9 of the exercise training group were not significantly different, compared with the control. This meta-analysis was synthesized with findings from recently-published RCTs, and provides health-care providers and AAA patients an alternative idea for AAA management besides medication.

## Data Availability

All data and materials are accessible to the public upon reasonable request.
